# Links between an Owner’s Adult Attachment Style and the Support-Seeking Behavior of Their Dog

**DOI:** 10.3389/fpsyg.2017.02059

**Published:** 2017-11-30

**Authors:** Therese Rehn, Andrea Beetz, Linda J. Keeling

**Affiliations:** ^1^Department of Animal Environment and Health, Swedish University of Agricultural Sciences, Uppsala, Sweden; ^2^Konrad Lorenz Research Station, Department of Behavioural Biology, University of Vienna, Vienna, Austria; ^3^Department of Special Education, Institut für Sonderpädagogische Entwicklungsförderung und Rehabilitation, University of Rostock, Rostock, Germany

**Keywords:** dog–human relationship, dog attachment, caregiving, emotional bonding, dog welfare

## Abstract

The aim of this study was to investigate if an owner’s adult attachment style (AAS) influences how their dog interacts and obtains support from them during challenging events. A person’s AAS describes how they perceive their relationship to other people, but it may also reflect their caregiving behavior, and so their behavior toward the dog. We measured the AAS of 51 female Golden retriever owners, using the Adult Attachment Style Questionnaire (ASQ), and observed the reactions of the dog-owner dyads in response to different challenging situations [visual surprise, auditory stressor and social stressors like a person approaching dressed as ghost or in coat, hat and sunglasses]. In addition, the dog was left alone in a novel environment for 3 min. Interactions between the dog and owner were observed both before and after separation. Spearman rank correlation tests were made (between owner AAS and dog behavior) and where correlations were found, Mann–Whitney *U*-tests were made on the dogs’ behavioral response between high and low scoring groups of owners of the different subscales of the ASQ. The more secure the owner (ASQ subscale ‘*Confidence’*), the longer the dog was oriented to the two sudden stressors (the visual and auditory stressor). The more anxious the owner (ASQ subscale ‘*Attachment anxiety*’), the longer the dog oriented toward the owner during the approach of the strange-looking person and the dog showed less lip licking during separation from the owner. The more avoidant the owner (ASQ subscale ‘*Avoidant attachment*’), the longer the dog oriented toward the owner during the visual stressor, the less it was located behind the owner during the auditory stressor and the less it was oriented toward the auditory stressor. These links between owner attachment style and dog behavior imply that dogs may develop different strategies to handle challenging situations, based on the type of support they get from their owner.

## Introduction

The relationship between dogs and owners has been suggested to resemble that between a child and its mother (e.g., Topál et al., 1998), where the dog is considered the attached individual in the relationship and the owner acts as an attachment figure. Dogs seem to elicit caregiving behavior in their owners (Kellert and Wilson, 1993; Askew, 1996; Archer, 1997; Nagasawa et al., 2015) and humans tend to interact with dogs and children in a similar way (Mitchell, 2001; Prato-Previde et al., 2006; German, 2015), although this may vary depending on e.g., the age of the dog and the context in which interactions occur (Julius et al., 2013).

How dogs seek support from their owner in a challenging situation is probably influenced by the owner’s caregiving strategy, as well by experience from their previous interaction history, although this has not been systematically documented yet. Human adult attachment styles (AAS; e.g., Mikulincer and Shaver, 2007) describe how a person relates to other people, which expectation the person has toward others in relationships and how they handle relationships in general (Hazan and Shaver, 1987). One important aspect is that the AAS also reflects how a person takes care of others, their caregiving strategy (Mikulincer and Shaver, 2007), hence affecting the development of the attachment style of their child (Zeanah et al., 1995; George and Solomon, 1996; Main, 2000). Moreover, it affects how the child interacts with the environment, such as dealing with external stressors (Barrett et al., 2005). For example, people with a more secure AAS are usually more responsive and sensitive to their child’s signals and needs (i.e., giving it more social support) which in turn affects the development of the child’s attachment system in the direction of attachment security (e.g., De Wolff and van IJzendoorn, 1997).

People with a *secure* (also often called autonomous/confident) AAS are comfortable with being dependent on others and easily get close to other people (Hazan and Shaver, 1987; Bartholomew and Horowitz, 1991; Crowell et al., 1999). Those with a more *avoidant* (or dismissive) AAS often feel discomfort in relationships, are not relaxed with being too close to others and may have difficulties trusting others. People with an *anxious*/ambivalent AAS are often worried about others being reluctant to get close, fearful of being abandoned, concerned that other people do not really care about them, and they are often viewed by others as clingy.

Findings from human psychology suggest that children of sensitive and responsive caregivers (usually associated with a secure AAS) handle a stressful situation better compared to those of parents with insecure AAS (avoidant and anxious). This is probably due to changes in the child’s behavior depending on the level of attention and comfort it receives from their main caregiver (George, 1996), but it could also be because children of parents with different AAS may be exposed to stressful situations more or less often. There is evidence that securely attached children show lower stress responses (physiologically) when accompanied by their caregiver (Julius et al., 2013). Stress regulation is probably influenced by early experience where abuse, neglect or non-sensitive caregiving have negative effects. Avoidant caregivers restrict support-seeking and acknowledgment of distress, whereas anxious-ambivalent caregivers show increased attention to negativity and heightened expression of distress (Kobak and Sceery, 1988). Edelstein et al. (2004) found associations between AAS, as measured in self-reports, and the observed responsiveness of parents toward their child when exposed to a challenging situation (inoculation procedure). In their study, more avoidant parents showed lower responsiveness when the child reacted with high distress to the procedure (see also Rholes et al., 1995). Other studies have found that the more insecure (both avoidant and anxious) a person’s AAS is, the less physical comfort they provide their child after a threatening and painful procedure (Goodman et al., 1997). When faced with a threat, more securely attached children have a greater attention flexibility (gaze shifts between stressor and attachment figure) compared to insecurely attached children (Main, 2000). Upon reunion with an attachment figure after a separation, which serves to activate the attachment system, secure children seek proximity with the parent and are comforted by the contact with them. Avoidant children respond to reunion by actively avoiding and ignoring the parent, while the ambivalent children may seek proximity, comfort, and physical contact with the parent, but usually without calming down, feeling comforted or deactivating their attachment behavior (Ainsworth et al., 1978).

Attachment behavior in dogs toward their owners has been studied by using, e.g., adapted versions of the Ainsworth’s Strange Situation Procedure (ASSP), which was originally developed to investigate attachment in children (e.g., Topál et al., 1998; Palmer and Custance, 2008). Such studies indicate that dogs express attachment behavior toward their owners, including aspects of secure base effects. Moreover, based on the behavior expressed in the ASSP, dogs could be divided into different groups resembling the secure-insecure attachment dimensions, further suggesting similarities between the relationship between child–parent and dog–owner (Topál et al., 1998). Some links between AAS and owner-dog interactions have been found in previous studies. For example, owners with a more secure AAS had dogs that showed a behavioral response in the ASSP more similar to that of securely attached children (Siniscalchi et al., 2013). Also more recently, it was found that owners scoring higher on insecure avoidant AAS were more likely to own dogs suffering from separation anxiety (Konok et al., 2015). Presumably these dogs perceive less social support from their owners or become overly dependent on them. Schöberl et al. (2016) found that physiological responses in dogs were affected by the adult attachment profile of the owner. The higher the owner scored on anxious attachment, the higher was the dog’s cortisol reactivity during the ASSP. Cimarelli et al. (2016) investigated owners’ ‘interaction style,’ very similar to the description of different parenting styles, and their dogs’ responses during challenging situations. Owners whose interaction style toward the dog was characterized as warm and enthusiastic, had dogs who sought proximity with the owner during the approach of a threatening person, while no such associations were found with owners who were described as ‘social supporters’ (owners who e.g., praised and petted the dog a lot during the challenging situation). They also found an age effect, with older people scoring higher in “owner warmth.”

In the current study, owners’ AAS were assessed via the Adult Attachment Style Questionnaire (ASQ), a self-report based measure of AAS. Moreover, dog-owner dyads were exposed to different types of challenging situations in order to activate the attachment system in the dog and to study their support-seeking behavior. We decided not to use the ASSP because of concerns about order effects and that it may not be sufficiently stressful to active the attachment system in well socialized, adult dogs (Rehn et al., 2013). We speculated that owner AAS, employed to indirectly assess their caregiving styles, would have similar effects on dogs as they would on children of parents with different AAS (Ainsworth et al., 1978; Main, 2000) and based on recent anthrozoology literature (Rehn and Keeling, 2016; Schöberl et al., 2016), the following hypotheses were formulated:

H1: Dogs of owners who were more *secure* in their AAS would alternate their gaze between stressor and owner (as an indicator of greater flexibility in attention shifts), initially stay in close proximity to the owner (use them as secure base) but then approach the stressor without problems toward the end of the test or within reasonable time (secure base effect: regulation via feeling of security and social support). During separation, signs of distress and proximity seeking behaviors (e.g., looking at the door where the owner disappeared) were expected, and that the dogs would show a positive response to reunion with the owner, and calm down within a relatively short period of time once the owner had returned. The behavior described above was expected to resemble that of a child with a secure attachment, usually having a sensitive caregiver who him/herself has a secure AAS.
H2: Dogs belonging to rather *anxious* owners were expected to “cling” to their owner during stressor application, looking more toward the owner and be unwilling to approach the stressor (i.e., taking longer to get in contact with the stressor). These dogs were expected to show more distress behavior (e.g., vocalization, escape attempts) during separation than the secure ones, and express an intense greeting behavior. Such dogs were not expected to calm down quickly when the owner returned. Their behavior therefore was expected to resemble that of a child with an insecure ambivalent style of attachment to their caregiver.
H3: Dogs belonging to rather *avoidant* owners were expected to look elsewhere (i.e., neither to the owner nor the stressor), preoccupying themselves with something not related to the stressor. These dogs were expected to show minor distress during separation from the owner, and the least intense greeting behavior when the owner returned. Their behavior therefore was expected to resemble that of a child with an insecure avoidant style of attachment to their caregiver.

## Materials and Methods

### Subjects and Test Locations

A homogenous group of participants was chosen in order to minimize confounding effects in the current study. In total, 51 female owners 48 ± 11.2 (mean ± standard deviation) years old (range: 22–72) and their dogs participated in the study. All dogs were Golden retrievers [25 males (of which 4 were neutered) and 26 females (of which 4 were neutered)]. The age of the male dogs was 4.7 ± 2.0 (2–9 years old) and the age of female dogs was 5.1 ± 1.6 (2–8 years old).

Tests were executed at five different geographical places in Sweden; Uppsala, Söderköping, Gothenburg, Ängelholm and Karlstad, during the autumn of 2014.

One female (TR) always took the role as the TL, and two other females were assisting or acted as the stressors during the tests (see below).

For the use of animals in research, an ethical permit was obtained from the animal ethics committee in Uppsala (reference number: C69/14). According to Swedish legislation (SFS 2003:460), no ethical approval was needed for human subjects, although all owners gave an informed written consent before participating in the study. The information on the written consent was approved by the Uppsala Ethics committee for the use of animals in research.

### Behavioral Tests

Dog–owner dyads participated in five different test situations. Dogs were assigned to the first four tests (referred to as ‘stressors,’ which were all executed outdoors) in a balanced order [William’s design (Williams, 1949), in blocks of four dogs]. During stressor application, owners were asked to stay neutral, follow our instructions and not to respond to the behavior of the dog. The four outdoor test situations selected as stressors in this study were from two Swedish standardized behavior assessments; the Dog mentality assessment (e.g., Svartberg and Forkman, 2002) and the Dog behavior and personality assessment (Svenska Kennelklubben [SKK], 2015), in a few cases with minor adjustments which are described further below. Two of these tests were composed of visual or auditory stimuli (non-social, sudden threats), while the other two included the approach of a person which was possibly experienced as threatening by the dog due to their looks and approach style (social stimuli, slowly increasing level of threat). The reason for choosing both non-social and social stressors was that dogs may differ in their perception of these distinct situations (Goddard and Beilharz, 1984, 1986; Hsu and Serpell, 2003). Hence, we aimed to include a variety of stressors to maximize the chances that they would be experienced as challenging by the dogs. After the four outdoor tests, dogs were left alone for 3 min in a novel indoor test arena where their separation and reunion behavior toward their owners were observed (referred to as the ‘separation and reunion test’). This additional test was performed as it is suggested to be an important measure of attachment in humans (e.g., Ainsworth et al., 1978) and dogs (Rehn et al., 2013; Rehn and Keeling, 2016). In addition to being randomly allocated to blocks of four, dog-owner dyads were tested independently from each other to minimize any possible order influences.

All tests were recorded using two Sony Handycam video cameras (HDR-CX130).

#### Auditory Stressor (AUD)

This test situation was executed in the same way as within the Dog mentality assessment. While the dog was walking with the owner on a short leash, a sudden loud noise (∼3 s long sound duration) was presented from the side (1.5 m away). The sound source, which was triggered by quickly sliding chains and casserole lids over a corrugated sheet, was hidden slightly behind branches with leaves alongside the path. The owner was instructed to stop immediately when the sound started, let go of the leash and to remain passive, looking toward the stressor (the sound source). Thereafter, the owner was instructed by the TL to act according to **Table 1A** depending on the response of the dog. As soon as the dog approached the stressor (i.e., was within 5 cm or in physical contact with it) at any point in the test procedure, the test was over and the owner was encouraged to approach and praise the dog.

**Table 1A:** Procedure for the Auditory stressor test.

Minute	Action (if dog had not approached the stressor)
00:15	Owner walks halfway toward stressor (0.75 m), stops and remains passive
00:30	Owner walks all the way up to stressor, stops and remains passive
00:45	Owner squats by stressor, talks to/calls the dog in an encouraging manner
01:00	Test is over

*If the dog had already approached to within 5 cm or was in physical contact with the stressor at any time into the test, the owner rewarded the dog and the test was over. If it had not approached the stressor, the owner acted according to the above timetable.*

#### Visual Stressor (VIS)

The dog walked on a short leash on the left side of the owner and the TL walked on the right side of the owner. When at a distance of 3 m, a board was raised from the ground in front of the dyad. The owner was instructed to stop (as did the TL) immediately, let go of the leash and to remain passive. Thereafter, the owner was instructed by the TL to act according to **Table 1B** depending on the response of the dog. As soon as the dog approached the stressor, i.e., was within 5 cm or in physical contact with it, the test was over and the owner was encouraged to approach and praise the dog. This test situation was executed in the same way as within the Dog behavior and personality assessment, but the board (visual surprise) was adapted slightly. Rather than being in the shape of the silhouette of the upper body part of a person, the board was a regular dark brown rectangle to avoid any social associations.

**Table 1B:** Procedure for the Visual stressor test.

Minute	Action (if dog had not approached the stressor)
00:30	Owner and test leader walk all the way up to stressor, stop and remain passive
00:45	Owner and test leader squat by stressor, talk to/call the dog in an encouraging manner
01:00	Test is over

*If the dog had already approached to within 5 cm or was in physical contact with the stressor at any time into the test, the owner rewarded the dog and the test was over. If it had not approached the stressor, the owner acted according to the above timetable.*

#### Ghost (G)

This test situation was executed in the same way as within the Dog mentality assessment, with the exception that in this study, there was only one person (instead of two) with a white sheet over them and so dressed as a ‘ghost’. This person approached the dyad from the front. During the test, the owner and dog were stationary (dog on a 2 m long leash), while the threatening person approached them slowly in a step-wise manner including six stops (each lasting for 10 s) starting at a distance of 25 m. The owner was instructed not to let go of the leash unless the dog wanted to increase the flight distance during the approach phase. When the ‘ghost’ was 5 m away from the dyad, she stopped, turned her back toward the dyad and showed her hands. The approach phase lasted for 2 min in total, after which the owner was instructed to let go of the leash and to remain passive, then to act according to **Table 1C** depending on the response of the dog. As soon as the dog approached the stressor, i.e., was within 5 cm or in physical contact with the ghost-looking person, the test was over and the owner was encouraged to approach and praise the dog.

**Table 1C:** Procedure for the Ghost test.

Minute	Action (if dog had not approached the stressor)
00:15	Owner walks halfway toward stressor (2.5 m), stops and remains passive
00:30	Owner walks all the way up to stressor and stands face-to-face with the ‘ghost,’ remains passive
00:45	Owner and ‘ghost’ start talking to each other, owner allowed to call the dog
01:00	Owner removes the hood from the ‘ghost’ while still talking to ‘ghost’ and dog
01:15	‘Ghost’ and owner call dog
01:30	Test is over. ‘Ghost’ removes the white sheet, to reveal her normal clothes, and walks away from the position together with owner, encouraging the dog to interact

*If the dog had already approached to within 5 cm or was in physical contact with the stressor at any time into the test, the owner rewarded the dog and the test was over. If it had not approached the stressor, the owner acted according to the above timetable.*

#### Approaching Person (AP)

This test was executed in exactly the same way as within the Dog behavior and personality assessment. Dog and owner were stationary during the test, the dog was on a 4 m long leash, held by the TL. The test started by a hidden person (25 m from the dyad) clapping her hands three times, then leaving the hiding place and becoming visible to the dog. The person was dressed in a long coat, wearing dark sunglasses and a slouch hat. When visible, she clapped her hands three times again in a stationary position. The person then started to approach the dyad slowly in a step-wise manner including five stops (each lasting for 5 s). At a distance of 6 m, the person stopped for 5 s, then turned around. The approach phase lasted for 1.5 min, and the dog was unleashed when the person had turned her back to the dyad, while the owner and TL remained passive. The owner was instructed to act according to **Table 1D** depending on the response of the dog. As soon as the dog approached the stressor, i.e., was within 5 cm or in physical contact with the person, the test was over and the owner was encouraged to approach and praise the dog.

**Table 1D:** Procedure for the Approaching Person test.

Minute	Action (if dog had not approached the stressor)
00:30	Owner and test leader walk all the way up to the stressor and stand face-to-face with the person at a close distance, remain passive
00:45	Owner talks to/calls the dog
01:00	The approaching person calls the dog, owner is passive
01:15	Test leader removes sunglasses and hat, the coat is removed and the person walks 5 m away from her original position
01:30	Test is over. The person squats down with the side of her body toward the dog, calling the dog

*If the dog had already approached to within 5 cm or was in physical contact with the stressor at any time into the test, the owner rewarded the dog and the test was over. If it had not approached the stressor, the owner acted according to the above timetable.*

The separation and reunion test was executed after the stressor tests, in an indoor test arena (3 × 3 m) which the dog had never been exposed to before. The TL, owner and dog walked in to the room where the arena was located and the owner was instructed about the procedure. The TL started the recordings and left the room unobtrusively. In order to study possible differences in the owners’ caregiving strategy, the owner was free to decide when and how to leave the arena and the room shortly after the TL had left. The dog was left alone in the arena for 3 min, after which the owner returned and greeted the dog, instructed to behave as she would normally in a similar situation. The owner and dog were left undisturbed for 1.5 min before the TL entered and the test was over.

### Adult Attachment Style

Prior to testing the dogs, the ASQ (Feeney et al., 1994) was used to evaluate the owners’ AAS in relationships with other humans. The ASQ has been translated into Swedish (Håkanson and Tengström, 1996). It consists of 40 statements rated on a 6-point scale. Items contribute to three or five different subscales and the internal reliability of the scales are acceptable. In their original sample, Feeney et al. (1994) reported Cronbach’s alphas between 0.76 and 0.84 for the different subscales. In a Swedish sample of 1631 women Cronbach’s alphas between 0.83 and 0.89 were reported for the three subscales used in the current study (Axfors et al., 2017). Test-retest correlations ranged from 0.65 to 0.84. In the current study, the three main subscales were used: the *Confidence* subscale assesses the extent to which individuals are confident about themselves and about their relationships with others (similar to the secure AAS, hence this latter term is used throughout the paper). More secure people find it easy to trust and get along with others, and they do not mind depending on others or having others depending on them. Those scoring high on the *Attachment anxiety* subscale tend to believe it is important that others like them, they worry that they will not measure up to others’ standards, and they usually worry about their personal relationships and the risk of being abandoned. People scoring high on the *Avoidant attachment* subscale tend to have difficulties in trusting or being dependent on others, or to have other people depend on them. They often believe that achievement is more important than relationships, and they place little importance on getting along with others.

### Behavior Analyzes

Videos from the behavior tests were analyzed using two standardized ethograms (**Tables 2, 3**), building upon experiences in previous studies (e.g., Beerda et al., 1998, 2000; Topál et al., 1998; Rehn and Keeling, 2011). In the stressor tests (AUD, VIS, G, and AP) the ethogram focuses on the position of the dog, the distance to the owner and the orientation of the dog. In the separation and reunion test, besides focusing on the location, orientation and main behavior of the dog, we noted those secondary behavior patterns such as lip licking, body shaking etc. suggested to be associated with the psychological state of the dog (e.g., Beerda et al., 1998, 2000; Rehn and Keeling, 2011). Videos were coded by one experienced observer using the data management software Interact (Mangold Professional, version 9).

**Table 2:** Ethogram stressor tests.

Category	Type	Description
Position	Side	The dog is located at the side of the owner (any body part)
	Behind	The dog (full body) is located behind the owner (i.e., the owner is located between the dog and the stressor)
Distance owner	Close	The dog is <5 cm of (or in contact with) the owner
	Within leash distance	The dog is 0.05–2 m away from the owner
	Away	The dog is >2 m away from the owner
Head direction	Toward stressor	The dog’s nose is directed toward the stressor
	Toward owner	The dog’s nose is directed toward the owner
	Toward other	The dog’s nose is directed elsewhere (not toward stressor or owner)
	Toward owner and stressor	The dog’s nose is directed toward the stressor and owner (only applicable when/if owner had approached the stressor)

**Table 3:** Ethogram separation and reunion test.

Category	Type	Description	Rec
Location	Entrance	Dog (main part of body) is located close to the gate wall	Instant 5 s
	Middle	Dog (main part of body) is located in the middle of the arena	Instant 5 s
	Away	Dog (main part of body) is located furthest away from the gate wall	Instant 5 s
Orientation	Owner	The dog’s nose is directed toward the owner	Instant 5 s
	Door/Gate	The dog’s nose is directed toward the gate/door	Instant 5 s
Main behavior	Lying alert	Dog is lying down with the head lifted from the floor	Instant 5 s
	Lying resting	The dog is lying down with its head in contact with the floor	Instant 5 s
	Lying on back	The dog is lying down with its back toward the floor	Instant 5 s
	Sitting	The dog is sitting down with its front legs extended and hind legs curved	Instant 5 s
	Standing	The dog is standing up on all four paws	Instant 5 s
	Walking/Running	The dog is moving around, either walking, trotting or galloping	Instant 5 s
	Jumping	The dog is standing on its hind legs	Instant 5 s
Physical contact	Initiated by owner	The owner initiates the physical contact with the dog	1/0 5 s
	Initiated by dog	The dog initiates the physical contact with the dog	1/0 5 s
Verbal contact	Initiated by owner	The owner is talking to the dog	1/0 5 s
	Whine	The dog is whining	1/0 5 s
	Growl	The dog is growling	1/0 5 s
	Bark	The dog is barking	1/0 5 s
	Howl	The dog is howling	1/0 5 s
Secondary behavior	Exploring	Motor activity directed toward any physical aspect of the environment, dog is sniffing/licking/manipulating something	1/0 5 s
	Escape attempt	The dog is pushing/scratching/jumping toward the wall of the arena	1/0 5 s
	Tail wagging	Repetitive wagging movements of the tail	1/0 5 s
	Head shake	The dog shakes its head from side to side	1/0 5 s
	Body shake	The dog shakes the whole body from side to side	1/0 5 s
	Body stretching	The dog is extending/stretching a part of or whole body	1/0 5 s
	Yawning	The dog opens its mouth widely and inhales	1/0 5 s
	Panting	An increased frequency of inhalation and exhalation with mouth open	1/0 5 s
	Grooming	The dog is cleaning its body surface by licking/nibbling/picking/rubbing/scratching etc	1/0 5 s
	Lip licking	Dog is snout licking, tongue visible	Continuous
	Oral movements	Dog is opening and closing its mouth/’smacking’ without tongue visible	Continuous

With regards to the stressor tests, instantaneous sampling (Martin and Bateson, 2007) every second was used, including location in relation to the owner and head direction (**Table 2**). The number of gaze alternations was calculated as the total number of times the dog turned its face/nose toward the stressor or the owner during the whole of the challenging situation (not only the immediate shifts between owner and stressor). Also, the latency to approach the stressor was measured. This was measured from the time of the start of the test until the dog had approached (<5 cm) or was in physical contact with the stressor.

In the separation and reunion test, location and behavior were analyzed using instantaneous sampling every 5 s, one/zero sampling and continuous recording (Martin and Bateson, 2007) (**Table 3**). Moreover, latency to first physical contact upon reunion was measured (the time from when the owner entered the test arena to first physical contact).

### Statistical Analyzes

Data were summarized as mean proportion of sample points per test during the stressors and as mean proportion of sample points before, during and after separation in the separation and reunion test.

To investigate possible links between owner AAS and the behavior of the dogs in the test situations non-parametric statistics were used since data were not normally distributed and transformations of the data to reach appropriate distributions were unsuccessful. Spearman rank correlations were calculated with SAS software (version 9.4, © 2002–2012, SAS Institute Inc., Cary, NC, United States). For the variables where correlations were found, Mann–Whitney *U*-tests were performed. For these *post hoc* tests, dog–owner dyads were divided into groups, based on how the owner scored on each subscale of the ASQ [higher (group ‘high’) or lower (group ‘low’) than the median, and respondents with a score equal to the median were excluded from the analysis]. This additional analysis acts to further investigate significant correlations between owner AAS and the responses of their dog, so minimizing the risks associated with multiple comparisons.

Comparisons of the dogs’ behavior between separation and reunion phases were investigated using Wilcoxon sign ranked tests, with dog as the dependent variable.

Since previous studies have shown associations between age and interaction styles (e.g., Cimarelli et al., 2016), Spearman rank correlations were calculated between age of the owner and the outcome of the ASQ subscales (owner AAS). Also, the age of the dog was tested for associations with behavior during the tests and the owner’s AAS (Spearman rank correlation). The sex of the dog was tested for effects on the behavior during tests and for associations with owner AAS (Mann–Whitney *U* tests).

## Results

In this section we first give some general information about the behavior of the dogs in the different tests, followed by results related to effects of dog age and sex. Thereafter we present the results of the correlations according to the AAS.

In general, orientation toward the stressors was a more common response by the dogs (see Supplementary Information, Table S1 for complete report) than orientating toward the owner. Dogs were most often located near (<2 m) and at the side of their owner during stressor application. Compared to the other outdoor tests, dogs were more often located behind their owner when the ‘ghost’ approached the dyad. As expected, dogs were more often located close to and more often oriented toward the door during separation than when reunited with the owner in the separation and reunion tests (Supplementary Information, Table S2), indicating that dogs were seeking proximity to the owner when left alone. Also, dogs were whining for more than 50% of the time when left alone in the room, which stopped when the owner came back. When reunited, owners initiated more contact than did the dogs. They also spoke to their dogs for almost 80% of the time. Lip licking increased among dogs when reunited with the owner, so did activity (walking/running) and panting.

### Dog Age and Sex and Their Effects on the Behavioral Response during the Tests

The older the dog was, the more it was oriented to its owner during the AP (*R*^2^ = 0.38, *p* = 0.007). Also, older dogs had a longer latency to approach the AP (*R*^2^ = 0.32, *p* = 0.02). Female dogs spent more time behind the owner during the VIS and AUD [VIS: males: 0.06 (0.00–0.18) (median (95% CI), females: 0.00 (0.00–0.00) χ^2^ = 4.09, *p* = 0.04; AUD: males: 0.04 (0.00–0.32), females: 0.00 (0.00–0.00), χ^2^ = 3.86, *p* = 0.05]. Female dogs spent more time at a longer distance (‘away’) from the owner during the AUD [males: 0.00 (0.00–0.34), females: 0.00 (0.00–0.00), χ^2^ = 3.88, *p* = 0.05], and were faster to approach the source of the sound [males: 13.00 (7.20–47.21), females: 5.00 (3.00–6.35), χ^2^ = 9.02, *p* = 0.003].

The younger the dog was, the more active (walking/running) it was during separation in the separation and reunion test (*R*^2^ = -0.37, *p* = 0.007). Also, younger dogs initiated more physical contact with their owner when reunited (*R*^2^ = -0.36, *p* = 0.01). Male dogs showed more lip licking during separation from the owner [males: 0.11 (0.08–0.22), females: 0.07 (0.03–0.12), χ^2^ = 5.77, *p* = 0.02], as well as at reunion [males: 0.53 (0.40–0.67), females: 0.41 (0.30–0.53), χ^2^ = 4.50, *p* = 0.03].

Neither age nor sex of the dog were correlated or affected by the AAS of the owner.

### Owner Age and Adult Attachment Style

There were no significant correlations between the age of the owner and any of the outcomes of the different subscales of the ASQ. The overall distribution of responses to the ASQ are presented in the Supplementary Information, Table S3.

### Secure Owners

Correlation tests showed that dogs belonging to owners scoring higher on the *confidence* subscale of ASQ were more oriented toward the VIS and AUD, were less oriented to the owner during the VIS and had a shorter latency to approach the VIS (**Table 4**). Moreover, these dogs were less likely to position themselves behind the owner during the G.

**Table 4:** Correlations between the subscales of the Adult Attachment Style Questionnaire (ASQ) and the behavioral responses of dogs during stressor application and the separation and reunion test.

	Secure attachment	Anxious attachment	Avoidant attachment
G: Located	**-0.38^∗∗^**		
behind owner	L: 0.29 (0.13–0.47)^∗∗∗^		
	H: 0.05 (0.00–0.12)		
AP: Oriented		**0.29^∗^**	
owner		L:0.01 (0.00–0.02)^∗^	
		H: 0.03 (0.02–0.05)	
VIS: Located		0.33^∗^	
close to owner		L: 0.00 (0.00–0.00)^t^	
		H: 0.00 (0.00–0.00)	
SR: Lip licking		**-0.29^∗^**	
during separation		L: 0.16 (0.11–0.26)^∗∗^	
		H: 0.08 (0.05–0.11)	
SR: Tail wagging		-0.28^∗^	
at reunion		L: 0.89 (0.74–0.95)^t^	
		H: 0.79 (0.64–0.89)	
AP: Located			**-0.41^∗∗^**
close to owner			L: 0.00 (0.00–0.00)^∗∗^
			H: 0.00 (0.00–0.00)
AUD: Located			**0.34^∗^**
behind owner			L: 0.00 (0.00–0.00)^∗^
			H: 0.04 (0.00–0.40)
VIS: Oriented	**0.35^∗^**	-0.28^∗^	
stressor	L: 0.38 (0.26–0.56)^∗^	L: 0.73 (0.41–1.00)^t^	
	H: 0.93 (0.39–1.00)	H: 0.35 (0.22–0.79)	
VIS: Latency to	-0.28^∗^		**0.32^∗^**
contact	L: 43.00 (10.00–51.17)^t^		L: 5.00 (3.20–6.80)^∗^
	H: 9.00 (5.00–43.39)		H: 14.00 (6.24–50.30)
AUD: Oriented	**0.30^∗^**		**-0.46^∗∗∗^**
stressor	L: 0.62 (0.49–0.76)^∗∗^		L: 0.88 (0.75–1.00)^∗∗∗^
	H: 0.83 (0.74–0.92)		H: 0.53 (0.47–0.70)
VIS: Oriented owner	**-0.46^∗∗∗^**	0.31^∗^	**0.33^∗^**
	L: 0.07 (0.05–0.13)^∗∗^	L: 0.00 (0.00–0.05)^t^	L: 0.00 (0.00–0.05)^∗∗^
	H: 0.00 (0.00–0.04)	H: 0.06 (0.005–0.12)	H: 0.07 (0.01–0.12)

*Figures in bold were significantly different in correlation tests and in the additional Mann–Whitney *U*-tests. N (Correlation tests) = 51, N (Confidence low) = 24, N (Confidence high) = 24, N (Attachment anxiety low) = 20, N (Attachment anxiety high) = 20, N (Avoidant attachment low) = 25, N (Avoidant attachment high) = 20. ^t^*P* ≤ 0.1; ^∗^*P* ≤ 0.05; ^∗∗^*P* ≤ 0.01; ^∗∗∗^*P* ≤ 0.001.*

*(VIS, visual stressor; AP, approaching person; AUD, auditory stressor; G, ‘Ghost’; SR, separation and reunion test), L = median in low scoring group (95% confidence interval), H = median in high scoring group (95% confidence interval).*

The additional group comparisons of the behavior of dogs whose owners scored low and high on the *confidence* subscale supported all these results with the exception of the finding that dogs with owners who scored high in confidence were slower to approach the VIS and so this result is not considered further.

### Insecure Anxious Owners

Owners scoring higher on the *attachment anxiety* subscale of the ASQ, had dogs who were oriented more toward the owner during the AP and the VIS as well as less oriented to the VIS (**Table 4**). Also, these dogs stayed closer to the owner during the VIS. Dogs belonging to owners with a more anxious AAS showed less lip licking during separation from the owner and less tail wagging upon reunion with the owner in the separation and reunion test.

The additional group comparisons of the behavior of dogs whose owners scored low and high on the *attachment anxiety* subscale supported that dogs of owners scoring high had dogs who were oriented more toward the owner during the AP and that these dogs showed less lip licking during separation, but only tended to support the other results.

### Insecure Avoidant Attachment

Owners scoring high on the *avoidant attachment* subscale had dogs who oriented more to their owner during the VIS and less toward the stressor during the AUD (**Table 4**). It took these dogs longer to approach the source of the VIS. These dogs were more often located behind the owner during the AUD, but were less often close to their owner during the AP.

The additional group comparisons of the behavior of dogs whose owners scored low and high on the *avoidant attachment* subscale supported all of these results.

## Discussion

In this study, links between owner AAS and the behavior of dogs in challenging situations were found. This supports that AAS may be used as an indirect measure of the person’s caregiving style and indicates provision of social support toward the dog. Since this was a correlation study, cause and effect are difficult to distinguish. However, it seems plausible that AAS is more stable than to be affected by the behavior or temperament of the dog (Schrafe and Bartholomew, 1994). In summary, dogs belonging to more secure owners were less likely to stand behind the owner during the approach of a potentially threatening person (the ‘ghost’), they were more oriented to the auditory and the visual stressors and less oriented to the owner during the visual stressor. Dog of more anxious owners were more oriented to the owner during the approach of a strange looking person and expressed less lip licking (possibly indicating lower stress) during separation from the owner. Dogs of more avoidant owners were less likely to stand close to the owner during the approach of a strange looking person, were behind the owner and less oriented toward the auditory stressor, but they were more oriented toward the owner during the visual stressor. In the following sections we discuss these results in the light of our predictions for the behavior of the dogs belonging to owners with each of the AASs. Our predictions were built upon knowledge from the literature of how children to parents with different AAS or caregiving styles behave, and how the children with different attachment styles themselves behave. Finally, we speculate on the possibility that these dogs may have different attachment styles, developed according to their owner’s AAS.

Our general observations of the dogs’ behavior during the tests revealed that dogs were often oriented to the stressors, and preferred to stay at the side of the owner rather than behind. This suggests that dogs did not use their owners as protection. In the separation and reunion tests, dogs expressed behaviors indicative of attachment to their owners. For example, dogs were seeking proximity to the owner during separation (e.g., being located near or oriented toward the door), they showed different levels of distress (by e.g., increased whining during separation) and they initiated contact with the owner at reunion, as well as wagged their tails more intensely. Perhaps surprisingly, exploration, which is a measure of the secure base effect of the owner, was not increased in the presence of the owner. However, the recording time of the reunion phase (1.5 min) may have been too short for the dogs to calm down after the negative experience of being left alone in the novel room. Panting was expressed as quite a high level, both during separation and afterward. Panting may indicate arousal, and according to our results this increased when the owner came back. Noteworthy though was, that the recordings were made during the summer, and the panting may have partly been due to temperature regulation. Lip licking, another indicator of positive (Rehn and Keeling, 2011) or negative (e.g., Beerda et al., 1998, 2000) arousal, was observed both during separation and at reunion. That it increased during reunion may indicate a positive emotional state when re-establishing contact with the owner or alternatively related to expressing friendly, submissive behavior in this social context.

Our predictions regarding the behavior of dogs belonging to more secure owners were not supported by our results. Dogs belonging to more secure owners looked more at the stressor during the challenging situations, rather than showing higher amounts of gaze alternations between the stressor and the owner which was in contrast to our hypothesis. This may reflect an owner who is supporting the dog’s attempts to independently deal with problems or threats, leading to more confidence also in the dog. This latter was further supported by the lower likelihood of these dogs positioning themselves behind the owner during the approach of the ‘ghost.’ The increased attention to the stressor may also reflect that dogs did not find their owners useful to refer to socially when challenged. However, this is not in line with evidence from human psychology, which suggests that secure children [who are likely to have a parent who is more secure (see e.g., van IJzendoorn, 1995)] are better able to shift their attention between threat and safe haven (the attachment figure) than are children with insecure styles (Emde, 1980; Main, 2000).

Our predictions regarding the behavior of dogs belonging to more anxious owners were partly supported by our results. Dogs participating with more anxious owners oriented longer toward their owner during stressor presentation. Indeed, it has been shown that dogs’ attentiveness to humans is affected, not only by familiarity (Mongillo et al., 2010), but also by the relationship quality (Topál et al., 1997; Horn et al., 2013). Previous studies suggest that the ‘closer’ the relationship (based on e.g., amount of joint activities and frequent feeding), the more the dogs gazed at its human demonstrator while he/she was manipulating a box under laboratory settings or when solving a problem (Topál et al., 1997; Horn et al., 2013). It has been argued that the closer proximity between individuals the greater propensity to acquire important information from each other (Swaney et al., 2001), while others suggest that it is the nature of past interactions that is more important when it comes to information gathering, at least in humans (Main, 2000). The findings in the current study, where dogs of more anxious owners gazed longer at the owner during stressor application are somewhat in line with the findings in human psychology. In children, insecure ambivalent individuals are less flexible in their attention during a stressful situation and seem to focus most on the safe haven, the attachment figure, compared to secure children (Main, 2000). Moreover, ambivalent children turned to and relied more on information provided by their parent than information provided by an unfamiliar person (Corriveau et al., 2009). Obviously, we cannot say anything about the dog’s ‘trust in their owner’ in the current study, but one could speculate that dogs tried to gather information about the stressor by looking at the owner. Among children, looking toward the caregiver has been proposed not only to reflect comfort seeking, but also the search for information about ambiguous or frightening stimuli (Walden and Kim, 2005; Stenberg and Hagekull, 2007).

During separation from their owners, dogs of more anxious owners showed less lip licking. Lip licking can be expressed in negative situations (Beerda et al., 2000) or when dogs are positively aroused (Rehn and Keeling, 2011; Rehn et al., 2014). Assuming separation was experienced as negative, dogs belonging to more anxious owners did not express their distress by showing a high frequency of lip licking. Schöberl et al. (2016) showed that owner AAS affected the physiological response of dogs during a challenging attachment test, where dogs of more anxious owners released higher levels of cortisol during the test compared to dogs of more secure owners. Behaviorally however, they did not express any differences in the test, rather it was found that the more active-excitable the dog was the lower their cortisol release was. Perhaps these dogs (showing less lip licking in the current study and higher cortisol release in Schöberl et al., 2016) are coping more passively during stressful events (De Boer et al., 1990; Korte et al., 1992). In humans, anxious mothers are usually unpredictable in their responsiveness to the child’s needs (Smith and Pederson, 1988; Isabella et al., 1989; Isabella and Belsky, 1991) and are least effective when they do respond to their child’s signals (Smith and Pederson, 1988), which may be related to these cautious/passive behavioral responses also among dogs, although they may be physiologically affected.

Our predictions regarding the behavior of dogs belonging to more avoidant owners were mostly supported by our results. It has been suggested that children of parents with an avoidant AAS focus their attention *away* from their parent in a stressful situation, but also away from the stressor (Main et al., 1985; Kirsh and Cassidy, 1997; Main, 2000). Dogs belonging to insecure avoidant owners in the current study actually did look less toward the auditory stressor, but more toward the owner during the visual stressor. However, these dogs located themselves less often close to or behind the owner during stressor application, indicating that they did not use owners as safe havens or secure bases.

Links between owner AAS and the behavior of their dogs were found, although, not surprisingly, not all of our hypotheses were supported in the current study. Two specific methodological details of our experimental design that may have influenced this are our instructions to the owners and our emphasis on quantitative scoring methods. In the current study owners were informed to stay neutral during the stimuli presentations (except for during the separation and reunion test). This could have affected the behavior of dogs in different ways. In a study where dogs were allowed to watch their owner’s emotional reaction to a novel object, dogs approached the object faster if the owner showed a positive emotional response to the stimulus and took longer to approach if owners reacted in a negative way (Merola et al., 2012). As owners in the current study did not respond as they would usually do in the challenging situation, dogs may have been ‘confused’ and reacted differently toward the owner than would they normally. On the one hand, the standardization of the owners’ behavior minimized the confounding effects of owner cues on the behavioral responses of the dogs to the stimuli, which is important. On the other hand, it meant we could not assess the owner’s caregiving style by observational data (apart from in the separation and reunion test) nor the possible bidirectional processes occurring during the threatening situations. As caregiving is influenced partly by the emotional reaction of the relationship partner (Grossmann et al., 1986; Simpson et al., 1992), future studies may consider to let the owner react naturally to the stressor and the dog. Secondly, the lack of links found between owner AAS and the dog’s behavior during reunion with the owner, may be explained by the choice of quantitative scoring methods. While enhancing the objectivity of the recordings, since they were according to the predetermined ethogram, this approach may miss subtle or unanticipated details of the mutual interactions. After our study was carried out, Solomon et al. (2014) and Schöberl et al. (2016) reported qualitative scoring systems for attachment styles in dogs, which seems a promising line for future investigations of attachment between dogs and humans. The lack of variety in the dogs’ behavior may also be related to the short observation period (1.5 min), since a lot of dogs did not seem to calm down within the given time frame. Also, the number of subjects participating was limited, which should be considered when interpreting and implementing the findings. Nevertheless, the sample in this study was homogenous (same breed and female owners) and the design balanced with standardized tests, which increases its reliability. According to our analyses in the current study, there were a few behavioral differences related to dog age and sex. This should be considered in future attachment studies. However, in the current study there were no associations between the dogs’ age or sex and the owners’ AAS. A final aspect to consider, although more difficult to correct for in future studies considering the aim is to look at attachment, is the fact that dog–owner dyads were not created randomly for this study. That is to say, owners came with their own dog who they presumably had chosen. Even when selecting a puppy, the owner at least had the possibility of matching the dog’s temperament to their own interest or attachment style. Perhaps longitudinal studies could investigate the extent to which initial choice of dog influences the links between owner AAS and dog behavior in challenging situations.

In summary, links between owner AAS and the behavior of their dogs in challenging situations were found. The responses among dogs were partly similar to what has been observed among parent-child dyads suggesting that it would be interesting to further evaluate the connections between observed caregiving behavior of the owner, AAS, and the attachment style of the dog.

## Author Contributions

All authors (TR, AB, and LK) have contributed to the design of the experiment. TR executed the practical work and was main responsible for doing the analyses, although AB and LK were included in deciding the approach. TR was main responsible in writing the first drafts of the manuscript, but AB and LK were involved in critically revising it to its current state. All authors (TR, AB, and LK) discussed and contributed to the interpretation of the results in the study. TR, AB, and LK all approve the final version of the manuscript to be published and agree to be accountable for ensuring that questions related to the accuracy or integrity of any part of the work are investigated and resolved.

## Conflict of Interest Statement

The authors declare that the research was conducted in the absence of any commercial or financial relationships that could be construed as a potential conflict of interest.

## Abbreviations

AAS: adult attachment style
AP: approaching person, dressed in a coat, hat and sunglasses
ASQ: adult attachment style questionnaire
AUD: auditory stressor
G: Ghost (person dressed as a ghost)
TL: test leader
VIS: visual stressor
