# Supplementary material for: Links between an Owner’s Adult Attachment Style and the Support-Seeking Behavior of Their Dog
Source: Front Psychol. 2017 Nov 30;8:2059. doi: 10.3389/fpsyg.2017.02059 (PMC5715226; doi:10.3389/fpsyg.2017.02059)
Supplement: Supplementary file 1 [file Table_1.docx]

Supplementary material

**Table 1.** Descriptive statistics of the dogs’ behavior during the outdoor test situations

| Test | Located at the side of owner^a^ | Located behind owner^a^ | Positioned near (<2m) owner^a^ | Positioned away (>2m) from owner^a^ | Latency to approach stressor^b^ | Oriented to stressor^a^ | Oriented to owner^a^ |
| --- | --- | --- | --- | --- | --- | --- | --- |
| **Auditory stressor** | 0.25 (0.24-0.29) | 0.00 (0.00-0.04) | 1.00 (0.90-1.00) | 0.00 (0.00-0.00) | 7 (5-11) | 0.75 (0.67-0.83) | 0.00 (0.00-0.00) |
| **Approaching person** | 0.06 (0.03-0.19) | 0.02 (0.008-0.04) | 0.94 (0.88-0.98) | 0.02 (0.00-0.05) | 138 (131-144) | 0.72 (0.63-0.77) | 0.02 (0.02-0.03) |
| **Ghost** | 0.54 (0.40-0.71) | 0.14 (0.07-0.27) | 0.87 (0.81-0.95) | 0.13 (0.05-0.19) | 180 (161-185) | 0.65 (0.61-0.75) | 0.03 (0.02-0.04) |
| **Visual stressor** | 0.48 (0.33-0.57) | 0.00 (0.00-0.06) | 0.80 (0.59-0.91) | 0.20 (0.09-0.36) | 27 (8-49) | 0.55 (0.35-0.80) | 0.04 (0.00-0.06) |

^a^Reported as median proportion of sample points per test (95% confidence interval)

^b^Reported as median s (95% confidence interval)
