# Supplementary material for: Links between an Owner’s Adult Attachment Style and the Support-Seeking Behavior of Their Dog
Source: Front Psychol. 2017 Nov 30;8:2059. doi: 10.3389/fpsyg.2017.02059 (PMC5715226; doi:10.3389/fpsyg.2017.02059)
Supplement: Supplementary file 2 [file Table_2.docx]

Supplementary material

**Table 2.** Descriptive statistics of the responses during the separation and reunion test

| Behavior^a^ | During separation | At reunion with owner | Separation vs. reunion |
| --- | --- | --- | --- |
| Located near door | 0.73 (0.61-0.92) | 0.26 (0.21-0.32) | Z^b^=15.5, *P*<0.0001 |
| Oriented to door | 0.64 (0.53-0.75) | 0.06 (0.05-0.11) | Z=24.5, *P*<0.0001 |
| Lying down (alert or resting) | 0.28 (0.14-0.51) | 0.00 (0.00-0.11) | Z=14.5, *P*<0.0001 |
| Walking/running | 0.06 (0.03-0.11) | 0.18 (0.11-0.23) | Z=-13.0, *P*<0.0001 |
| Exploring | 0.06 (0.03-0.08) | 0.00 (0.00-0:00) | Z=15.0, *P*<0.0001 |
| Whining | 0.54 (0.32-0.64) | 0.00 (0.00-0.00) | Z=20.0, *P*<0.0001 |
| Lip licking | 0.10 (0.08-0.14) | 0.44 (0.39-0.56) | Z=-21.5, *P*<0.0001 |
| Panting | 0.16 (0.06-0.36) | 0.88 (0.78-0.90) | Z=-23.5, *P*<0.0001 |
| Tail wagging | 0.00 (0.00-0.03) | 0.79 (0.74-0.89) | Z=-24.5, *P*<0.0001 |
| Oriented to owner | N.A.^c^ | 0.58 (0.53-0.67) | N.A. |
| Dog initiate physical contact | N.A. | 0.39 (0.28-0.50) | N.A. |
| Owner initiate physical contact | N.A. | 0.79 (0.56-0.74) | N.A. |
| Owner verbal contact | N.A. | 0.77 (0.68-0.84) | N.A. |
| Latency to physical contact (s) | N.A. | 4.0 (3.0-6.0) | N.A. |

^a^Behaviors are reported as median proportion of sample points (95% confidence interval) per phase (separation and reunion), except for latency to physical contact which is reported as median s.

^b^Z= Wilcoxon sign rank test statistics

^c^N.A.= Not applicable
