# Supplementary material for: Links between an Owner’s Adult Attachment Style and the Support-Seeking Behavior of Their Dog
Source: Front Psychol. 2017 Nov 30;8:2059. doi: 10.3389/fpsyg.2017.02059 (PMC5715226; doi:10.3389/fpsyg.2017.02059)
Supplement: Supplementary file 3 [file Table_3.docx]

Supplementary material

**Table 3.** Descriptive statistics of the owners’ responses to the subscales of the Adult Attachment Style Questionnaire

| Adult Attachment Style Questionnaire subscale | Median (95 % confidence interval) |
| --- | --- |
| Secure attachment (Confidence) | 4.75 (4.50-5.00) |
| Anxious attachment | 2.38 (2.23-2.69) |
| Avoidant attachment | 2.94 (2.69-3.06) |
